# Wing Geometric Morphometrics as a Tool for the Identification of *Culex* Subgenus Mosquitoes of *Culex* (Diptera: Culicidae)

**DOI:** 10.3390/insects11090567

**Published:** 2020-08-25

**Authors:** Roseli França Simões, André Barretto Bruno Wilke, Carolina Romeiro Fernandes Chagas, Regiane Maria Tironi de Menezes, Lincoln Suesdek, Laura Cristina Multini, Fabiana Santos Silva, Marta Gladys Grech, Mauro Toledo Marrelli, Karin Kirchgatter

**Affiliations:** 1 Institute of Tropical Medicine, School of Medicine, University of São Paulo, São Paulo, SP 05403-000, Brazil; rosefs@usp.br (R.F.S.); lincoln.suesdek@butantan.gov.br (L.S.); fabinss30@gmail.com (F.S.S.); mmarelli@usp.br (M.T.M.); 2 Department of Public Health Sciences, Miller School of Medicine, University of Miami, Miami, FL 33136, USA; axb1737@med.miami.edu; 3 Institute of Ecology, Nature Research Centre, Vilnius 08412, Lithuania; crfchagas@gmail.com; 4 Applied Research Department, Zoological Park Foundation, São Paulo, SP 04301-905, Brazil; 5 Department of Specialized Laboratories, Superintendence for Endemic Disease Control, SUCEN, São Paulo, SP 01027-000, Brazil; rmtironi@gmail.com; 6 Butantan Institute, São Paulo, SP 05503-900, Brazil; 7 Department of Epidemiology, School of Public Health, University of São Paulo, São Paulo, SP 01246-904, Brazil; lauramultini@usp.br; 8 Centro de Investigación Esquel de Montaña y Estepa Patagónica (CIEMEP), CONICET and UNPSJB, Facultad de Ciencias Naturales y Ciencias de la Salud, Sede Esquel, Esquel 9200, Chubut, Argentina; mgrech@comahue-conicet.gob.ar

**Keywords:** *Culex*, mosquitoes, morphometry, Atlantic Forest, Patagonia

## Abstract

**Simple Summary:**

Different mosquito species have different ecology and behaviors. Therefore, the correct identification of vector mosquito species is essential for the development of targeted mosquito control operations. Traditionally, the identification of mosquitoes to species relies on differences in their external morphological characters. Identifying mosquitoes can be challenging if the specimen is either damaged or if only a few morphological characters can be used to sort them apart. For this reason, this study focused on the use of wing geometric morphometrics to identify *Culex* species from the subgenus *Culex* that are not easily identified by their external morphology. We analyzed the wing shape variation of 11 different species. Our results indicated that the species in this study were identified with high degrees of confidence based on their wing shape variation. From all possible comparisons in the cross-validated reclassification test, 87 yielded values higher than 70%, with 13 comparisons yielding 100% reclassification scores. Overall, our results are suggesting that wing geometric morphometrics is a reliable tool to identify *Culex* species of the subgenus *Culex*.

**Abstract:**

*Culex* is the largest subgenus within the genus *Culex* that includes important vectors of diseases. The correct identification of mosquitoes is critical for effective control strategies. Wing geometric morphometrics (WGM) has been used to identify mosquito species alongside traditional identification methods. Here, WGM was used for eleven *Culex* species from São Paulo, Brazil, and one from Esquel, Argentina. Adult mosquitoes were collected using CDC (Centers for Disease Control) traps, morphologically identified and analyzed by WGM. The canonical variate analysis (CVA) was performed and a Neighbor-joining (NJ) tree was constructed to illustrate the patterns of species segregation. A cross-validated reclassification test was also carried out. From 110 comparisons in the cross-validated reclassification test, 87 yielded values higher than 70%, with 13 comparisons yielding 100% reclassification scores. *Culex* *quinquefasciatus* yielded the highest reclassification scores among the analyzed species, corroborating with the results obtained by the CVA, in which *Cx*. *quinquefasciatus* was the most distinct species. The high values obtained at the cross-validated reclassification test and in the NJ analysis as well as the segregation observed at the CVA made it possible to distinguish among *Culex* species with high degrees of confidence, suggesting that WGM is a reliable tool to identify *Culex* species of the subgenus *Culex*.

## 1. Introduction

Vector-borne diseases (VBD) constitute a significant burden on human society, with millions of people infected every year. Moreover, there is growing evidence indicating that VBDs are responsible not only for the morbidity and mortality associated with the acute infection of the diseases they cause, such as malaria, dengue, and yellow fever but also for persistent long-term morbidity in the form of severe neurologic complications and fetus malformations associated to Zika virus infections [1,2,3].

Mosquitoes from the *Culex* genus are considered primary vectors for many pathogens, including different arboviruses (Table 1). It is widely accepted that controlling mosquitoes is the most effective way to prevent VBDs. However, controlling mosquitoes is a complex task that relies on multi-sectoral collaborations between local government, scientists, and the community, in which, many critical steps must logically build on each other in order to achieve effective mosquito control [4,5]. Effective mosquito control strategies have to take into account and base their action on the targeted mosquito vector and develop the control actions based on its ecology and behavior. In this context, the correct identification of mosquito species is critical for planning and guiding effective long-term mosquito control operations.

The correct identification of mosquitoes is exceptionally challenging, mainly because there is a lack of evident anatomical differences in many species and the distinguishing features are often restricted to male genitalia, in addition to the fact that the number of taxonomists is dwindling. *Culex* is by far the largest genus of tribe Culicini (Diptera: Culicidae: Culicinae) with 768 species allocated to 26 subgenera [6]. The subgenus *Culex* by itself comprises 198 species with unique ecology and behavior that should be taken into account for control actions [6]. Identification of mosquitoes is predominantly carried out through the taxonomic key based on the external morphology, and to a lesser extent by molecular techniques [7,8,9,10].

Both the traditional morphological and molecular mosquito identification methods have advantages and disadvantages. Morphological identification is inexpensive and quick and can be done in the field, but since the correct identification relies on morphological structures, damaged mosquitoes often cannot be identified. Moreover, cryptic species and species complexes are, in some cases, indistinguishable using only their morphology. Molecular identification of mosquitoes does not require intact specimens, only a fraction of the body is enough to provide reliable identification. However, this technique is expensive and requires a specialized laboratory.

Wing geometric morphometrics (WGM) has been successfully used to correctly identify mosquito species [11,12,13,14], being proven an efficient tool to be used alongside traditional morphological and molecular mosquito identification. It is relatively inexpensive and only requires that at least one wing is intact. The identification of mosquitoes can be made in the field in a rudimentary lab equipped only with a stereo microscope, a digital camera, and a computer.

Mosquito wings are especially suitable to be used for mosquito identification due to its bidimensional characteristic and wide availability of anatomical landmarks that can be used to distinguish species [15]. Here we hypothesize that the variation of the anatomical landmarks based on wing venation will make it possible to successfully distinguish among the species of the *Culex* subgenus of *Culex*. Therefore, the objective of this study was to use WGM to identify 11 species of the *Culex* subgenus.

## 2. Materials and Methods

### 2.1. Mosquito Sampling and Identification

**Figure 1:**
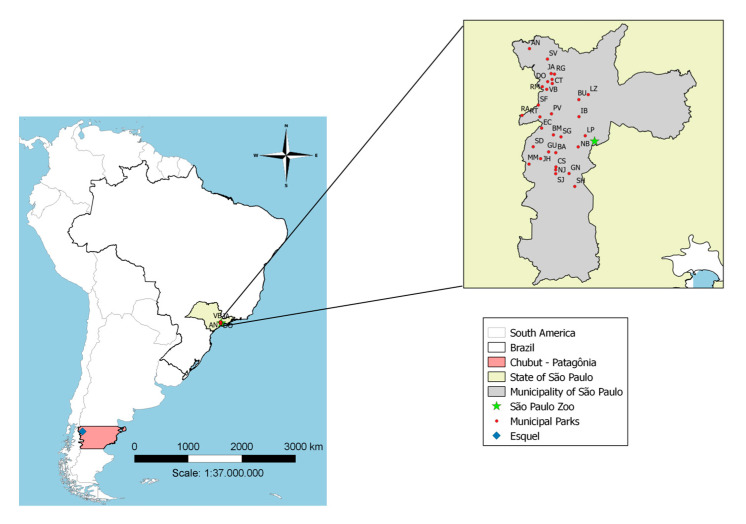
Collection sites. Anhanguera (AN), Barragem (BA), Burle Marx (BM), Buenos Aires (BU), Castelo (CS), Cidade Toronto (CT), São Domingos (DO), Eucaliptos (EC), Ganhembu (GN), Guarapiranga (GU), Ibirapuera (IB), Jacinto Alberto (JA), Jardim Felicidade (JF), Jardim Herculano (JH), Lina e Paula Raia (LP), Luz (LZ), M. Boi Mirim (MM), Nabuco (NB), Nove de Julho (NJ), Previdência (PV), Cohab Raposo Tavares (RA), Rodrigo de Gasperi (RG), Vila dos Remédios (RM), Raposo Tavares (RT), Santos Dias (SD), Colina de São Francisco (SF), Severo Gomes (SG), Shangrilá (SH), São José (SJ), Senhor do Vale (SV), Orlando Villas-Boas (VB), São Paulo ZOO (✩), and Esquel—Argentina (□).

Mosquitoes were collected in three different sites: (i) Municipal parks on the city of São Paulo, Brazil: Anhanguera (23°25′05.8″ S, 46°46′56.1″ W), Barragem (23°40′40.1″ S, 46°42′59.0″ W), Burle Marx (23°38′00.2″ S, 46°43′20.6″ W), Buenos Aires (23°32′43.7″ S, 46°39′31.5″ W), Castelo (23°42′48.2″ S, 46°42′57.6″ W), Cidade Toronto (23°30′19.1″ S, 46°43′30.7″ W), São Domingos (23°30′02.3″ S, 46°44′12.4″ W), Eucaliptos (23°36′59.5″ S, 46°45′06.5″ W), Ganhembu (23°43′47.2″ S, 46°40′59.5″ W), Guarapiranga (23°40′33.1″ S, 46°44′03.7″ W), Ibirapuera (23°35′16.9″ S, 46°39′30.3″ W), Jacinto Alberto (23°28′49.4″ S, 46°43′41.0″ W), Jardim Felicidade (23°29′42.2″ S, 46°43′32.5″ W), Jardim Herculano (23°41′33.9″ S, 46°45′15.7″ W), Lina e Paula Raia (23°38′08.2″ S, 46°38′34.1″ W), Luz (23°31′58.8″ S, 46°38′06.9″ W), M. Boi Mirim (23°42′22.8″ S, 46°47′00.1″ W), Nabuco (23°39′48.1″ S, 46°39′37.4″ W), Nove de Julho (23°43′14.1″ S, 46°43′00.4″ W), Previdência (23°34′51.1″ S, 46°43′37.9″ W), Cohab Raposo Tavares (23°35′05.5″ S, 46°48′03.3″ W), Rodrigo de Gasperi (23°28′54.9″ S, 46°43′10.7″ W), Vila dos Remédios (23°30′47.9″ S, 46°45′00.7″ W), Raposo Tavares (23°35′18.5″ S, 46°45′22.3″ W), Santos Dias (23°39′46.8″ S, 46°46′22.6″ W), Colina de São Francisco (23°33′32.4″ S, 46°45′36.1″ W), Severo Gomes (23°38′18.2″ S, 46°42′12.3″ W), Shangrilá (23°45′43.4″ S, 46°40′08.2″ W), São José (23°43′48.9″ S, 46°43′00.1″ W), Senhor do Vale (23°26′39.8″ S, 46°44′14.3″ W), Orlando Villas-Boas (23°31′11.5″ S, 46°44′20.7″ W) [16,17]; (ii) Zoo in the city of São Paulo (Fundação Parque Zoológico de São Paulo—FPZSP) (23°39′03″ S, 46°37′14″ W)[18]; and (iii) in a forest-steppe ecotone near Esquel city, Southern Argentina (42°55′00.0” S, 71°21′00.0″ W) (Figure 1).

The collections were carried out with the use of CDC (Centers for Disease Control) Miniature light traps [19] baited with CO_2_ (dry ice). The CDC traps were installed 1.5 m above the ground before down for 12 h. All collected mosquitoes were identified using taxonomic keys [9,20], then the right wing of each specimen was detached from the body and mounted between a microscope slide and coverslip with Canada balsam (Sigma-Aldrich, St. Louis, MO, USA) (Table 1).

This study was performed according to the Ethical Principles in Animal Research. It was approved by the Ethics Committee of Institute of Tropical Medicine, University of Sao Paulo (CPE-IMT/193 and CPE-IMT/371A), and the Brazilian Ministry of Environment (SISBIO 34605-4). The datasets analyzed during the current study are available in the Mendeley Data repository (https://data.mendeley.com/datasets/nzsxjnng63/1).

### 2.2. Data Acquisition and Morphometrics Analysis

**Figure 2:**
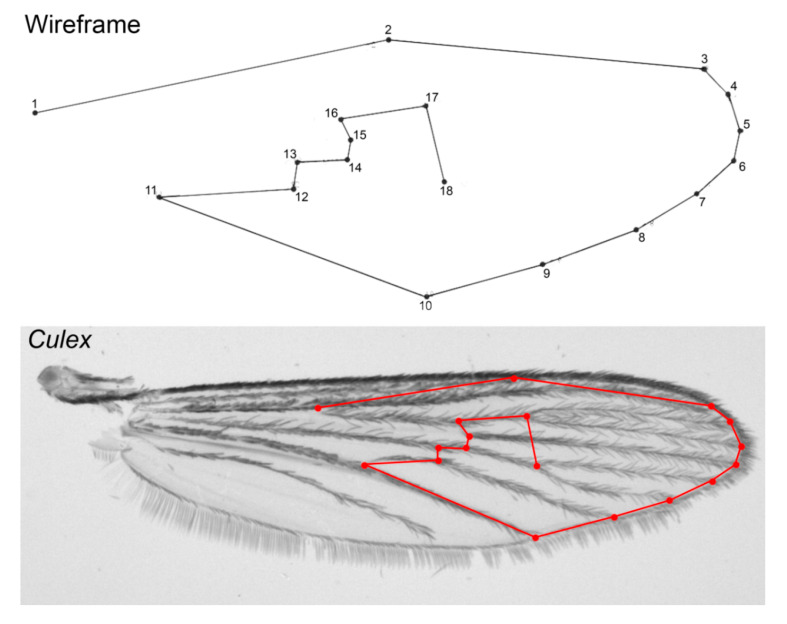
Right wing of *Culex* specimen showing the 18 landmarks used in this study.

Each wing was photographed under 40× magnification with a Leica DFC320 digital camera (Leica Camera AG, Solms, Germany) coupled to a Leica S6 stereomicroscope (Leica Camera AG, Solms, Germany), and subsequently, 18 type II landmarks were digitized by one of the authors (RFS) using TpsDig V1.40 software [29], as in Wilke et al. [14] (Figure 2). The selected landmarks are homologous and can be found in all representatives of the Culicidae family [14].

We calculated the allometry (influence of the wing size in the wing shape) by multivariate regression of the Procrustes coordinates against centroid size using a permutation test with 10,000 randomizations. Discriminant analysis in a morphospace defined by a canonical variate analysis (CVA) was performed to determine the degree of dissimilarity between populations and to calculate the Mahalanobis distances. All specimens were reclassified according to its wing similarity to the average shape of each group (group 1 vs. group 2 and group 2 vs. group 1) using cross-validation tests based on Mahalanobis distances. All the analyses were carried out in MorphoJ 1.02 [30]. Neighbor-Joining (NJ) trees were constructed to display the Mahalanobis distances between populations using PAST 1.89 [31] with 1000 bootstrap replicates (30 specimens of *Aedes aegypti* were used as outgroup).

## 3. Results

The allometry was minor but significant 0.72% (*p* = 0.031) and was not removed from the analysis since the influence of size in the shape of wings were considered evolutionarily relevant and, thus, can be informative for the species identification process.

**Figure 3:**
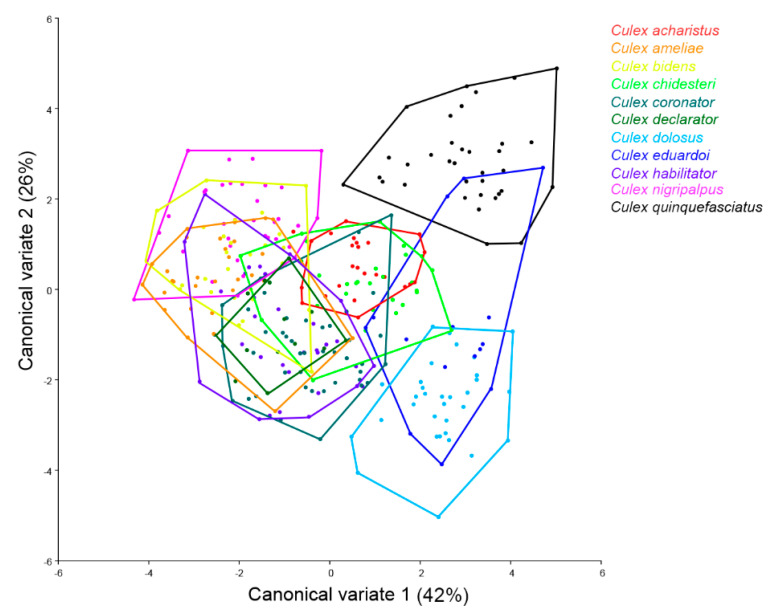
Morphological space produced by the first two canonical variates for *Culex* species based on 18 wing landmarks.

The results from the CVA for all *Culex* species displayed substantial segregation from *Cx. quinquefasciatus* from all other species, being partially overlapped solely with *Cx. eduardoi*. Similar results were also found for *Cx. dolosus*, partially overlapping with *Cx. eduardoi* and in a much lesser extent to *Cx. chidesteri*. *Culex nigripalpus* was completely segregated from *Cx. acharistus*, *Cx. coronator*, *Cx. quinquefasciatus*, *Cx. eduardoi,* and *Cx. dolosus*, and was only partially overlapped with *Cx. chidesteri*. *Culex acharistus* greatly overlapped with *Cx. chidesteri* and *Cx. declarator*, and to a lesser extent to *Cx. habilitator* and *Cx. eduardoi* (Figure 3).

**Figure 4:**
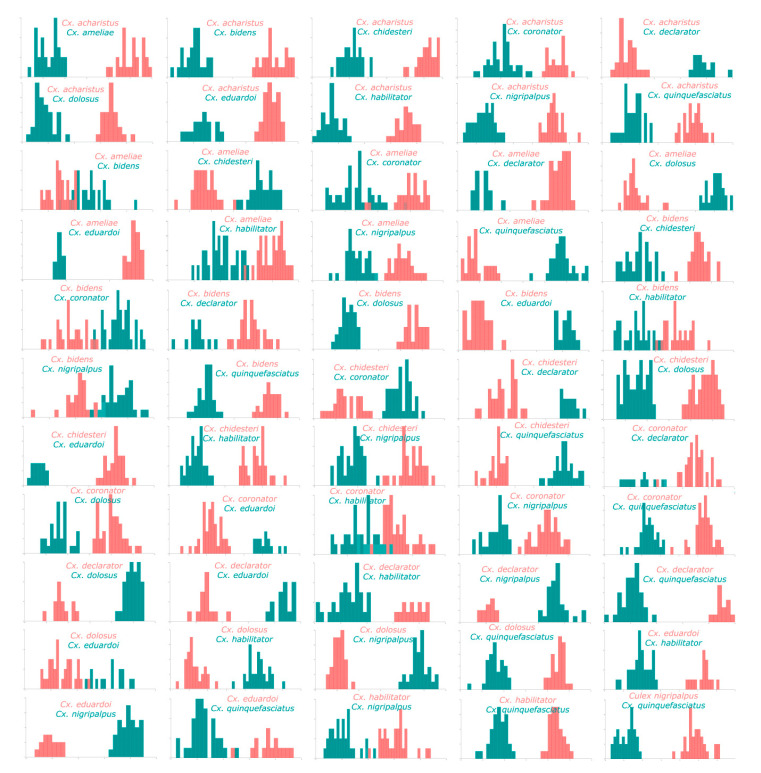
Wing shape diagram of the first canonical variable from the pairwise comparison for *Culex* species based on 18 wing landmarks. X-axis: first canonical variable; Y-axis: frequency.

A pairwise CVA analysis for all species showed that from the 55 possible comparisons, none presented major wing shape pattern overlaps and only 12 had minor overlaps (Figure 4).

**Figure 5:**
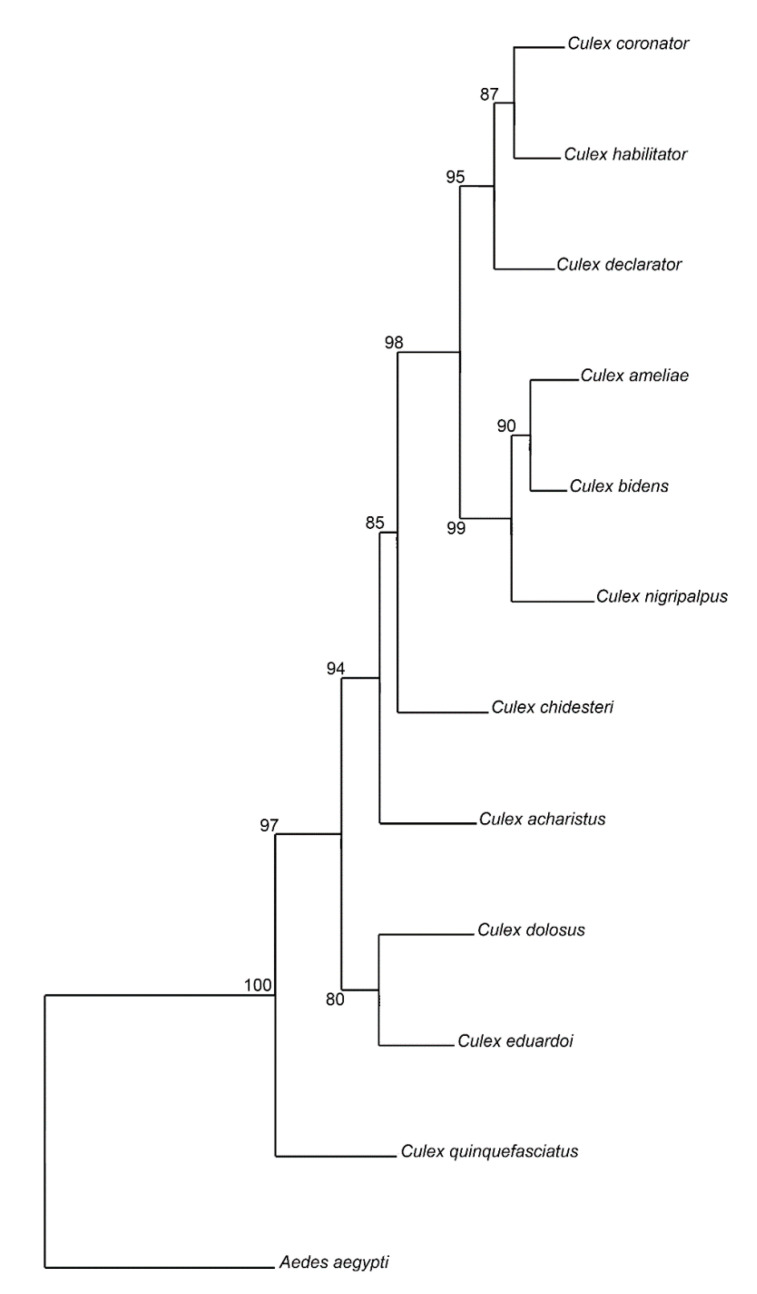
Neighbor-Joining tree for *Culex* species based on Mahalanobis distances with 1000 bootstrap replicates. *Aedes aegypti* (*n* = 30) was used as an outgroup.

The NJ tree analysis resulted in high bootstrap values (>80) for all species (Figure 5). *Culex quinquefasciatus* yielded the highest value (100), being segregated from all other species in a single branch. *Culex nigripalpus*, *Cx. declarator* and *Cx. acharistus* were also segregated in single branches with high bootstrap values (>94). Some species cluster together with high bootstrap values (>95): *Cx. dolosus* and *Cx. eduardoi*, *Cx. ameliae* and *Cx. bidens,* and *Cx. coronator* and *Cx. habilitator* (Figure 5).

The cross-validated reclassification results were high, with the overall mean value of 82% accuracy (Table 2). From the 110 possible tests, 13 achieved 100% accuracy, 45 were higher than 90% and only six yielded values below 50% accuracy. *Culex quinquefasciatus* yielded the highest scores in the cross-validated reclassification test, with an average of 94% accuracy, followed by *Cx. dolosus* with 89% accuracy and *Cx. acharistus* with 87%. *Culex declarator* had the lowest reclassification average, yielding an average of 73% accuracy. The lowest reclassification scores were obtained by the comparison between *Cx. bidens* vs. *Cx. ameliae* yielding 44% accuracy. The comparison between *Cx. declarator* vs. *Cx. acharistus*, *Cx. declarator* vs. *Cx. bidens*, and *Cx. habilitator* vs. *Cx. declarator* yielded 46% accuracy scores.

## 4. Discussion

The species phylogeny and taxonomy of the *Culex* genus is intricate and has been a matter of debate over the last decades [32]. Microevolutionary and speciation processes add an increased level of complexity to the correct identification of *Culex* species [33]. Cryptic species, such as the species complex formed by *Cx. quinquefasciatus* and *Cx. pipiens* and the *Cx. coronator* complex, pose as an increasing need for additional taxonomic tools for the correct identification of *Culex* species [34,35].

Here, we were able to identify 11 sympatric species from the *Culex* subgenus with high degrees of confidence using WGM technique based on quantitative analyses of mosquito wing venation characters. It is important to note that, with the exception of *Cx. quinquefasciatus*, all *Culex* species used in this study are not easy to recognize even for entomologists with some experience. However, cryptic species were not analyzed here. Although WGM is also a very useful tool for identifying populations, in some cases it is unable to find clearly differentiating patterns [36] and this can also happen with cryptic species, in which, to obtain an accurate identification, the use of WGM alongside other taxonomical techniques, substantially increasing the accuracy scores, can be necessary.

In this study, we analyzed 73% (11/15) of the *Culex* subgenus species collected in the study areas [16,17,18]. Considering that all analyses were made for species from the same subgenus and therefore a reduced variation would be expected, the classification power of the WGM analyses was consistently reliable. Both the cross-validated reclassification test and the bootstrap values from the NJ analysis resulted in high values, even when compared to the ones found for species from different genera [14]. The results obtained by the cross-validated reclassification test were high, with the overall mean value of 82% accuracy. From all comparisons, approximately 50% of the results from the cross-validated reclassification tests yielded values above 90%. Furthermore, considering that the lowest reclassification average among all comparisons was found for *Cx. declarator* (73% accuracy), and only a few punctual comparisons resulted in values lower than 50% we strongly believe these results are reliable enough to be used in routine mosquito control operations.

In many studies of interspecific variation, WGM has been used to identify species based on the reclassification scores. Analysing three *Aedes* species from Thailand, values of 73 to 93% were found [37] and in two *Haemagogus* species from Brazil, values were from 67 to 81% [38]. Still in Brazil, *Cx. quinquefasciatus* and *Cx. nigripalpus* could be distinguished by wing shape with accuracy rates ranging from 85 to 100% [39] and for four *Culex (Culex)* species from Argentina, re-classification was 100% in *Cx. bidens* and *Cx. mollis*, 89% in *Cx. interfor* and 94% in *Cx. tatoi* [13]. Cross-validated reclassification showed that WGM is an effective analytical method to distinguish between *Anopheles (Kerteszia) bellator*, *An. cruzii* and *An. homunculus* with a reliability rate varying from 78 to 88% [40]. However, when larger numbers of species were analyzed together (11 *Anopheles* species from Colombia), like here in this study, reclassification scores were quite lower, reaching >80% for six species and >75% for eight species, but with scores of 46%, 66%, and 69%, for *An. benarrochi*, *An. oswaldoi* and *An. strodei* [41].

The lowest bootstrap value on the NJ analysis was 80 for the *Cx. dolosus* and *Cx. eduardoi*. These species are notably challenging to identify by traditional morphologic characters since specimens in the adult life stage are difficult to distinguish and larval stages have only a few morphological differences that can be used for their correct identification [32]. The females of *Cx. dolosus* and *Cx. eduardoi*, sister to one another in the NJ tree, are also morphologically similar. Very few morphological characters differentiate them: The presence of basal white bands in the abdominal tergites, pleural integument with dark areas, and yellowish scales antealar patches, which also distinguish them from other species of the subgenus *Culex*. Moreover, it is worth considering that the smaller size of *Cx. eduardoi* sample (*n* = 15) and its high heterogeneity in the morphospace may be limiting factors for interpretations involving this species.

Females of *Cx. bidens* with legs without evident clear marking, as in the case of this study, present morphological characters very similar to *Cx. ameliae*. Appendices of the head, parts of the abdomen and thorax are similar, in both species, in terms of its scales. Such similarities result in greater difficulty in separating these species, most likely explaining the proximity between them in the NJ tree. However, in this study, the morphological differentiation of these females was possible due to the appressed scales present on the occiput.

The proximity of the *Culex* females of the Coronator complex to the females of *Cx. habilitator* in the NJ tree could be explained mainly in the case of *Cx. habilitator* presenting well-marked tarsi similar to the *Cx. coronator*. In this condition, the two species present morphological similarities related to the covering of scales of appendices of the head, such as the maxillary and proboscis palps and parts of the shield on the thorax. Most of the females from *Cx. habilitator*, collected for the elaboration of this article, presented tarsi little marked with clear color, a characteristic that includes these specimens in the taxonomic key together with *Cx. pseudojanthinosoma*, and not with *Culex* from the Coronator complex, in dichotomy with *Cx. scimitar*. However, it is a single character. Other similar characters for *Cx. habilitator* and *Cx. pseudojanthinosoma* species refer to the scales in the proboscis and thorax, being the same as those of the Coronator complex.

*Culex quinquefasciatus* and *Cx. nigripalpus* are the primary vectors for West Nile virus (WNV), Eastern Equine encephalitis (EEE), and lymphatic filariasis (LF) [42,43,44,45,46,47]. Together, these diseases affect millions of people worldwide and endanger almost half of the world’s population [48,49]. *Culex quinquefasciatus* and *Cx. nigripalpus* can be found in high numbers in urban environments. Developing countries that have undergone chaotic urbanization processes often lack basic sanitation, resulting in polluted rivers and untreated sewage. These features are intensely explored by *Culex* mosquitoes which are able to thrive in such conditions, in which they feed on blood on widely available human hosts and oviposit their eggs in highly polluted breeding habitats, absent from predators and abundant in organic matter [4,5,8,9,50].

*Culex* subgenus species are also capable of transmitting avian malaria to birds of various species throughout the globe including endangered species [51]. Many studies have demonstrated full or partial (part of the life cycle has not been demonstrated experimentally) vectorial competence of different *Culex* (*Culex*) spp. transmitting avian *Plasmodium* spp. such as *Cx. annulirostris*, *Cx. annulus*, *Cx. antennatus*, *Cx. gelidus*, *Cx. nigripalpus*, *Cx. pipiens*, *Cx. pseudovishnui*, *Cx. salinarius*, *Cx. sitiens*, *Cx. stigmatosoma*, *Cx. tarsalis*, *Cx. tritaeniorhynchus*, *Cx. univittatus*, *Cx. restuans,* and *Cx. quinquefasciatus* [50]. However, only *Cx. nigripalpus* and *Cx. quinquefasciatus* are listed among the 25 different *Culex* (*Culex*) species reported in Brazil (http://www.mosquitocatalog.org/). Previous studies from our group have detected avian malaria transmission in the São Paulo Zoo [52]. In this study, we used five *Culex* (*Culex*) species collected in this active transmission site. However, so far, there is no record in the literature of *Plasmodium* spp. detected in any of these five species. Although the analysis to test these mosquito species for *Plasmodium* presence is still in progress, considering that these mosquitoes were collected in a short period of time, we recommend that further research be conducted to enable long-term mosquito sampling and screening. Thus, in addition to identifying all *Culex* subgenus species that are present in a given area, it is essential to detect the *Plasmodium* infection in these mosquitoes to precisely establish their roles in the transmission of avian malaria. The correct identification of *Culex* mosquitoes to species level is critical to enable the better mapping of infection risks, the development of more effective mosquito control strategies, and increase our knowledge on the evolutionary relationships of host-pathogen interactions.

Even though more studies using WGM to identify mosquito species are available some obstacles still have to be overcome before this technique can be used in routine mosquito control operations. The further development and maintenance of reliable databases would greatly help to develop and validate WGM guidelines and protocols directly impacting its usefulness and effectiveness in correctly identifying mosquito species on a global scale. Moreover, additional validation using more specimens from each species, as well as more species, are needed to increase the identification reliability and guide future WGM efforts in the identification of mosquito vectors. However, in its current form, WGM identification standards can be considerably helpful to identify species of which the traditional identification is difficult. WGM can also be used alongside traditional and molecular techniques serving as an option rather than an antagonizing strategy.

## 5. Conclusions

The results obtained in this study suggest the WGM technique is a reliable tool to identify *Culex* species of the subgenus *Culex*. The high values obtained at the cross-validated reclassification test and in the NJ analysis as well as the segregation observed at the canonical variate analysis made it possible to distinguish among the *Culex* species used in this study with high degrees of confidence for most specimens.

## Figures and Tables

**Table 1:** Collection data and epidemiological importance of sampled mosquito species.

Taxon	N *	Collection Site	Collection Year	Epidemiological Importance
*Culex (Cux.) acharistus* Root	26	Esquel	2016/2017	EEEV [21]
*Culex (Cux.) ameliae* Casal	30	FPZSP	2015	Unknown
*Culex (Cux.) bidens* Dyar	27	FPZSP	2015	EEEV [21]
*Culex (Cux.) chidesteri* Dyar	30	Municipal Parks of São Paulo City **	2010/2011	Unknown
*Culex (Cux.) coronator* Dyar and Knab	43	FPZSP	2015	SLEV [22], VEEV [23], WNV [24]
*Culex (Cux.) declarator* Dyar and Knab	13	FPZSP	2015	SLEV [25], *Dirofilaria immitis* [26]
*Culex (Cux.) dolosus* (Lynch Arribálzaga)	32	Municipal Parks of São Paulo City **	2010/2011	Unknown
*Culex (Cux.) eduardoi* Casal and Garcia	15	Municipal Parks of São Paulo City **	2010/2011	Unknown
*Culex (Cux.) habilitator* Dyar and Knab	32	FPZSP	2015	WNV [27]
*Culex (Cux.) nigripalpus* Theobald	34	Municipal Parks of São Paulo City **	2010/2011	EEEV [22], EVEV [22], KEYV [22], ROCV [22], SLEV [22], TENV [22], VEEV [22], WNV [24]
*Culex (Cux.) quinquefasciatus* Say	57	Municipal Parks of São Paulo City **	2010/2011	CHIKV [22], EEEV [22], MAYV [28], OROV [22], ROCV [22], SLEV [22], VEEV [22], WNV [22], ZIKV [22], *Wuchereria bancrofti* [9], *Dirofilaria immitis* [26]

* Number of females used in this study; ** [16,17]. Fundação Parque Zoológico de São Paulo (FPZSP) [18]; Eastern Equine Encephalitis Virus (EEEV), Saint Louis Encephalitis Virus (SLEV), Venezuelan Equine Encephalitis Virus (VEEV), West Nile Virus (WNV), Chikungunya Virus (CHIKV), Everglades virus (EVEV), Keystone Virus (KEYV), Mayaro Virus (MAYV), Oropouche Virus (OROV), Rocio Virus (ROCV), Zika Virus (ZIKV), and Tensaw (TENV).

**Table 2:** Results of pairwise cross-validated species reclassification tests (%).

Group 2												
		*Culex acharistus*	*Culex ameliae*	*Culex bidens*	*Culex chidesteri*	*Culex coronator*	*Culex declarator*	*Culex dolosus*	*Culex eduardoi*	*Culex habilitator*	*Culex nigripalpus*	*Culex quinquefasciatus*
**Group 1**	***Culex acharistus***	-	90	89	89	83	46	97	73	91	88	91
	***Culex ameliae***	88	-	44	83	74	77	100	100	66	74	91
	***Culex bidens***	84	57	-	79	81	46	100	87	69	62	94
	***Culex chidesteri***	92	87	96	-	93	62	91	80	94	91	89
	***Culex coronator***	88	83	67	72	-	62	84	67	47	88	91
	***Culex declarator***	64	80	48	59	81	-	94	73	59	82	94
	***Culex dolosus***	100	93	93	90	91	77	-	53	97	100	94
	***Culex eduardoi***	72	97	81	86	91	92	63	-	81	94	71
	***Culex habilitator***	96	73	67	86	58	46	88	67	-	85	94
	***Culex nigripalpus***	100	77	63	79	81	85	100	93	78	-	97
	***Culex quinquefasciatus***	92	100	100	100	91	100	100	60	100	97	-

Values below the diagonal correspond to mosquitoes from group 1 compared with group 2 and correctly identified. Each pairwise comparison results in two values, one for species A in comparison to species B and then species B in comparison to species A; values above the diagonal correspond to mosquitoes from group 2 compared with group 1 and correctly identified. *p*-value (parametric): <0.0001.
